# Anticancer therapy within the last 30 days of life: results of an audit and re-audit cycle from an Australian regional cancer centre

**DOI:** 10.1186/s12904-020-0517-3

**Published:** 2020-01-27

**Authors:** Mike Nguyen, Sean Ng Ying Kin, Evonne Shum, Alysson Wann, Babak Tamjid, Arvind Sahu, Javier Torres

**Affiliations:** 1 Austin Health, Melbourne, Australia; 2 Goulburn Valley Health, Shepparton, Australia; 3 Peninsula Health, Melbourne, Australia

**Keywords:** Quality of care, Aggressiveness, Immunotherapy, Chemotherapy, Mortality, Palliative care, Audit, Service improvement

## Abstract

**Background:**

The therapeutic landscape in medical oncology continues to expand significantly. Newer therapies, especially immunotherapy, offer the hope of profound and durable responses with more tolerable side effect profiles. Integrating this information into the decision making process is challenging for patients and oncologists. Systemic anticancer treatment within the last thirty days of life is a key quality of care indicator and is one parameter used in the assessment of aggressiveness of care.

**Methods:**

A retrospective review of medical records of all patients previously treated at Goulburn Valley Health oncology department who died between 1 January 2015 and 30 June 2018 was conducted. Information collected related to patient demographics, diagnosis, treatment, and hospital care within the last 30 days of life. These results were presented to the cancer services meeting and a quality improvement intervention program was instituted. A second retrospective review of medical records of all patients who died between 1 July 2018 and 31 December 2018 was conducted in order to measure the effect of this intervention.

**Results:**

The initial audit period comprised 440 patients. 120 patients (27%) received treatment within the last 30 days of life. The re-audit period comprised 75 patients. 19 patients (25%) received treatment within the last 30 days of life. Treatment rates of chemotherapy reduced after the intervention in contrast to treatment rates of immunotherapy which increased. A separate analysis calculated the rate of mortality within 30 days of chemotherapy from the total number of patients who received chemotherapy was initially 8% and 2% in the re-audit period. Treatment within the last 30 days of life was associated with higher use of aggressive care such as emergency department presentation, hospitalisation, ICU admission and late hospice referral. Palliative care referral rates improved after the intervention.

**Conclusion:**

This audit demonstrated that a quality improvement intervention can impact quality of care indicators with reductions in the use of chemotherapy within the last 30 days of life. However, immunotherapy use increased which may be explained by increased access and a better risk benefit balance.

## Background

There have been significant recent developments in the treatment of cancer with many new treatments demonstrating clinical evidence for improved survival and quality of life (QOL). Systemic anticancer therapy (SACT) now includes cytotoxic chemotherapy, endocrine or hormonal agents, targeted or biologic agents and immune checkpoint inhibitors. Non-chemotherapy treatments are often associated with simpler routes of administration, less but not negligible adverse effect profiles and the potential of profound and durable clinical responses. This has made the decision making process for commencing, continuing and ceasing SACT more complex and requires a careful consideration of key factors, specifically disease biology, patient and family expectations, and clinician biases.

Earle et al. [1] have proposed several indicators for the assessment of quality of care near the end of life including the rate of chemotherapy administration; emergency department (ED) presentation, hospitalisation and intensive care unit (ICU) admission; and lack of or late referral to palliative care and hospice services. Over the last few decades, there is a trend towards more aggressive care with US registry studies finding an increase in patients receiving chemotherapy within 14 days of death; and increased rates of ED presentation, hospitalisation and ICU admission in the last month of life [2]. Use of chemotherapy is associated with higher rates of cardiopulmonary resuscitation and mechanical ventilation, late hospice referral, death in ICU, and death in a non-preferred place [3]. In the current context of immune checkpoint inhibitors, use near the end of life is associated with poorer performance status, lower hospice enrolment and higher rates of death in hospital [4].

The rationale for SACT with palliative intent is primarily to improve or maintain quality of life. Despite this objective, the quality of life of patients as assessed by psychological and physical distress in the final week of life has been found to not improve in patients with moderate or poor performance status who received chemotherapy and in fact worsens in patients with good or excellent performance status who received chemotherapy [5].

There is increasing evidence of the benefit of early involvement of palliative care for patients with cancer. The seminal randomised control study by Temel and colleagues [6] demonstrated early palliative care consultation for patients with non small cell lung cancer (NSCLC) improved QOL, mood and depressive symptoms, and survival by more than two months. A secondary analysis of this study demonstrated that palliative care did not affect the number of chemotherapy regimens administered, but that chemotherapy near the end of life was reduced and hospice enrolment was higher [7]. Both the American Society of Clinical Oncology (ASCO) and the European Society of Medical Oncology (ESMO) have made recommendations in clinical practice guidelines for the concurrent use of SACT and early involvement of palliative care services for patients with advanced cancer [8, 9].

The first large scale report of mortality within 30 days of chemotherapy emanated from the National Confidential Enquiry into Patient Outcome and Death (NCEPOD) conducted in the United Kingdom [10]. The reported rate of mortality within 30 days of SACT was 2% and has become the historical benchmark. Subsequently, several centres have published data related to SACT within the last 30 days of life [4, 11–28]. A selection of studies are summarised in Table 1 with focus on recent publications and the Australasian context. Comparisons between these studies are difficult for several reasons; studies differed with regard to: the included and excluded tumour types, treatment with curative and palliative intent, and treatment modality. Only three reports included immune checkpoint inhibitors. In addition, the studies reported different outcome measures most commonly the number of deaths within 30 days of treatment as a proportion of all patients who received treatment and, less commonly, as the number of deaths within 30 days of treatment as a proportion of all deaths.

**Table 1:** Summary of studies reporting systemic anticancer therapy near the end of life

Author	Country	Population studied	Treatment included	Treatment rate within last 30 days of life
Gilsch 2019 [4]	USA	Deaths of patients who received immune checkpoint inhibitors	I	27%
Ang 2018 [11]	New Zealand	Patients who received SACT	CT T I	5.2%
Burgers 2018 [12]	The Netherlands	Patients with stage III or IV lung cancer treated with SACT	CT	6.2% (within 30 days of first cycle of chemotherapy)
GIlbar 2018 [13]	Australia	Patients who received SACT	CT T	5.6%
Hiramoto 2018 [14]	Japan	Deaths of patients who received SACT with palliative intent	CT	16.7%
Massa 2018 [15]	Italy	Patients with metastatic colorectal cancer who received SACT	CT	7.1% (last 14 days of life)
Dasch 2017 [16]	Germany	Inpatient deaths of patients with cancer	CT	38.3%
Falchook 2017 [17]	USA	Patients with metastatic lung, colorectal, breast, pancreas and prostate cancer	CT	10.1–14.1% (within the last 14 days of life)
Kraut 2017 [18]	USA	Deaths of patients with cancer	CT	6–16%
Wilson 2017 [19]	New Zealand	Patients who received SACT	CT	2.2%
Wallington 2016 [20]	UK	Patients with lung cancer who received SACT	CT T	8%
		Patients with breast cancer who received SACT	CTT	2%
Wein 2016 [21]	Australia	Deaths of patients managed with palliative intent	CT E T	26%
Khoja 2015 [22]	UK	Deaths of patients who received SACT	CT T I	4%
Pacetti 2015 [23]	Italy	Deaths of patients who received SACT with palliative intent	CT	24.3%
Philip 2015 [24]	Australia	Metastatic non small cell lung cancer	CT	1% (last 14 days of life)
Andelkovic 2013 [25]	Australia	Patients who received SACT	CT T	6.9%
Zdenkowski 2013 [26]	Australia	Patients who received SACT with palliative intent	CT T	12.2%
Yoong 2012 [27]	Australia	Patients who received SACT	CT T	3.4%
Kao 2009 [28]	Australia	Deaths of patients managed with palliative intent	CT	10%
Mort 2008 [10]	UK	Patients who received SACT	CT	2%

Figure Legends: CT - chemotherapy, T - targeted therapy, I - immune checkpoint inhibitor, E - endocrine / hormonal therapy

Subsequent to NCEPOD, Christie Cancer Centre in the United Kingdom implemented its key recommendation to review all deaths within 30 days of SACT at a morbidity and mortality meeting and reassess progress through an audit process. Over a four year period, this practice did not reduce the rate of deaths within 30 days of SACT and had a minor but statistically insignificant reduction in the rate of treatment related deaths [22]. In contrast, Wilson et al. reported two audits performed at Auckland Hospital six years apart [19]. Mortality within 30 days of treatment with chemotherapy fell minimally with rates of 2.8% in 2009 and 2.2% in 2015. They proposed a series of clinical interventions that have informed this improvement implementation plan.

The aim of the study was to identify the rates of SACT within the last 30 days of life at the institution in order to compare with published benchmarks. We examined the use of the different types of SACT to observe any changing trends in practice given the development of new therapies, especially in the contemporary paradigm of immune checkpoint inhibitors. The audit also assessed other quality of care and aggressiveness of care parameters. Results from the initial audit informed the implementation of a service improvement plan which was then followed by a re-audit to assess any effect this improvement plan had on clinical practice. This study is novel in the emerging era of immune checkpoint inhibitors and contributes to our understanding of quality use of SACT, aggressiveness of care near the end of life and institution based interventions to improve the quality of patient care.

## Methods

### Data collection

Data collected included age, gender, tumour type, performance status, intent of treatment, modality of systemic anticancer treatment, number of previous treatment lines, date of last treatment, date of death, date of referral to palliative care or hospice service, number of emergency department presentations, number of hospital admissions and number of intensive care unit admissions within the last 30 days of life. Systemic anticancer treatment was defined as cytotoxic chemotherapy, endocrine or hormonal treatments, targeted or biologic agents and immune checkpoint inhibitors.

### Initial data collection

A retrospective review was conducted of medical records for all patients managed at the Goulburn Valley Health oncology department who died between 1 January 2015 and 30 June 2018.

### Improvement implementation plan

The following improvement implementation plan was enacted.

- Results were presented to the local cancer services educational meeting to an audience comprising medical oncologists, other medical staff, chemotherapy unit nurses, research nurses, specialist cancer support nurses and palliative care health professionals. This was conducted within a week of the conclusion of the audit period.
- Palliative care and community hospice services contact details were collated into a single resource and distributed to medical oncologists and other clinicians. This was conducted within a week of the conclusion of the audit period.
- Discussion at weekly departmental meeting of all patients being considered for anticancer therapy with performance status Eastern Cooperative Oncology Group (ECOG) score 3 or greater; or cases of concern for any other reason
- All clinicians to assess and record patient’s performance status at commencement of anticancer treatment and at each subsequent outpatient clinic review
- Review of all cases of anticancer treatment within the last 30 days of life at monthly departmental mortality meeting
- Commitment to repeat audit in order to assess improvement

### Repeat data collection

A retrospective review was repeated of medical records for all patients managed at the Goulburn Valley Health oncology department who died between 1 July 2018 and 31 December 2018.

### Statistical analysis

The data was analysed using descriptive statistical techniques.

## Results

### Audit

#### Patient characteristics

In the initial audit period, there were 440 patients analysed. Patient characteristics are summarised in Table 2. 60% were male. The average age was 72.5 years. Only 11% had a haematological diagnosis. Patients with performance status ECOG score 0 or 1 comprised 23%, ECOG 2 40% and ECOG 3 or 4 38% of the total. The most common diagnoses were lung (87 patients), colorectal (62 patients), breast (42 patients), prostate (40 patients) and pancreas (34 patients) (Table 6).

**Table 2:** Characteristics of patients in the the audit period

	All Patients		Treatment within last 30 days		No treatment within last 30 days	
	Number	Percent	Number	Percent	Number	Percent
	440		120	27%	320	73%
Sex						
Male	263	60%	67	56%	196	61%
Female	177	40%	53	44%	124	39%
Discipline						
Oncology	392	89%	103	26%	289	74%
Hematology	48	11%	17	35%	31	65%
Average age (years)	72.5		71		73.3	
Performance status						
ECOG 0 or 1	99	23%	34	28%	65	20%
ECOG 2	175	40%	44	37%	131	41%
ECOG 3 or 4	166	38%	42	35%	124	39%
Line of treatment						
Never treated	89	20%			89	28%
First line	170	39%	59	49%	111	35%
Second line	108	25%	36	30%	72	23%
Third line or greater	73	17%	25	21%	48	15%

#### Anticancer treatment

Details of treatments are described in Table 2 and Table 3. 20% of patients had not received any anticancer therapy and managed solely with best supportive care. 39% had received one line of treatment, 25% had received two lines of treatment and 17% had received three or more lines of treatment. 120 patients of the total 440 deaths (27%) had received anticancer treatment within the last 30 days of life.

**Table 3:** Treatment and aggressiveness of care in the audit period

	All Patients		Treatment within last 30 days		No treatment within last 30 days	
	Number	Percent	Number	Percent	Number	Percent
Last treatment type						
Chemotherapy	243		66	27%	177	73%
Targeted/biologic	47		20	43%	27	57%
Endocrine/hormonal	53		25	47%	28	53%
Immunotherapy	30		14	47%	16	53%
Treatment intent						
Curative	19	4%	5	4%	14	4%
Palliative	421	96%	115	96%	306	96%
Parameters for aggressiveness of care						
Palliative care referral	272	65%	67	58%	205	67%
Palliative care referral beyond last 30 days	178	42%	32	28%	146	48%
More than one emergency presentations	43	10%	21	18%	22	7%
More than one hospitalisation	49	12%	20	17%	29	9%
Hospitalisation 14 or more days	60	14%	13	11%	47	15%
ICU admission	16	4%	7	6%	9	3%

66 of 243 patients (27%) whose last anticancer treatment was chemotherapy received chemotherapy within the last 30 days of life. This indicator was higher with the other treatment modalities: 43% in targeted / biologic agents, 47% in endocrine / hormonal agents and 47% in immune checkpoint inhibitors. Of the total number of patients who received chemotherapy, treatment within the last 30 days of life represented 8% of patients.

#### Aggressiveness of care

421 (96%) were treated with palliative intent. Of these, 65% of patients had a referral to palliative care or community hospice services. Referral was often late with 58% of referrals made within the last 30 days of life. Receiving treatment within the last 30 days of life when compared with not, was associated with higher rates of late palliative care referral (72% compared with 52%), more than one ED presentation (18% vs 7%), more than one hospital admission (17% vs 9%) and ICU admission (6% vs 3%).

### Re-audit

#### Patient characteristics

The re-audit period comprised 75 patients as summarised in Table 4. 53% were male. The average age was 70 years. Only 13% had a haematological diagnosis. Patients with performance status ECOG score 0 or 1 comprised 40%, ECOG 2 39% and ECOG 3 or 4 21% of the total. The most common diagnoses were colorectal (14 patients), lung (10 patients), prostate (7 patients), breast (6 patients), upper GI (5 patients) and melanoma (5 patients) (Table 6).

**Table 4:** Characteristics of patients in the the re-audit period

	All Patients		Treatment within last 30 days		No treatment within last 30 days	
	Number	Percent	Number	Percent	Number	Percent
	75		19	25%	56	75%
Sex						
Male	40	53%	10	53%	30	54%
Female	35	47%	9	47%	26	46%
Discipline						
Oncology	65	87%	19	29%	46	71%
Hematology	10	13%	0	0%	10	100%
Average age (years)	70.1		66.9		71.1	
Performance status						
ECOG 0 or 1	30	40%	4	21%	26	46%
ECOG 2	29	39%	7	37%	22	39%
ECOG 3 or 4	16	21%	8	42%	8	14%
Line of treatment						
Never treated	6	8%			6	11%
First line	25	33%	3	16%	22	39%
Second line	25	33%	10	53%	15	27%
Third line or greater	19	25%	6	32%	13	23%

#### Anticancer treatment

**Fig. 1:**
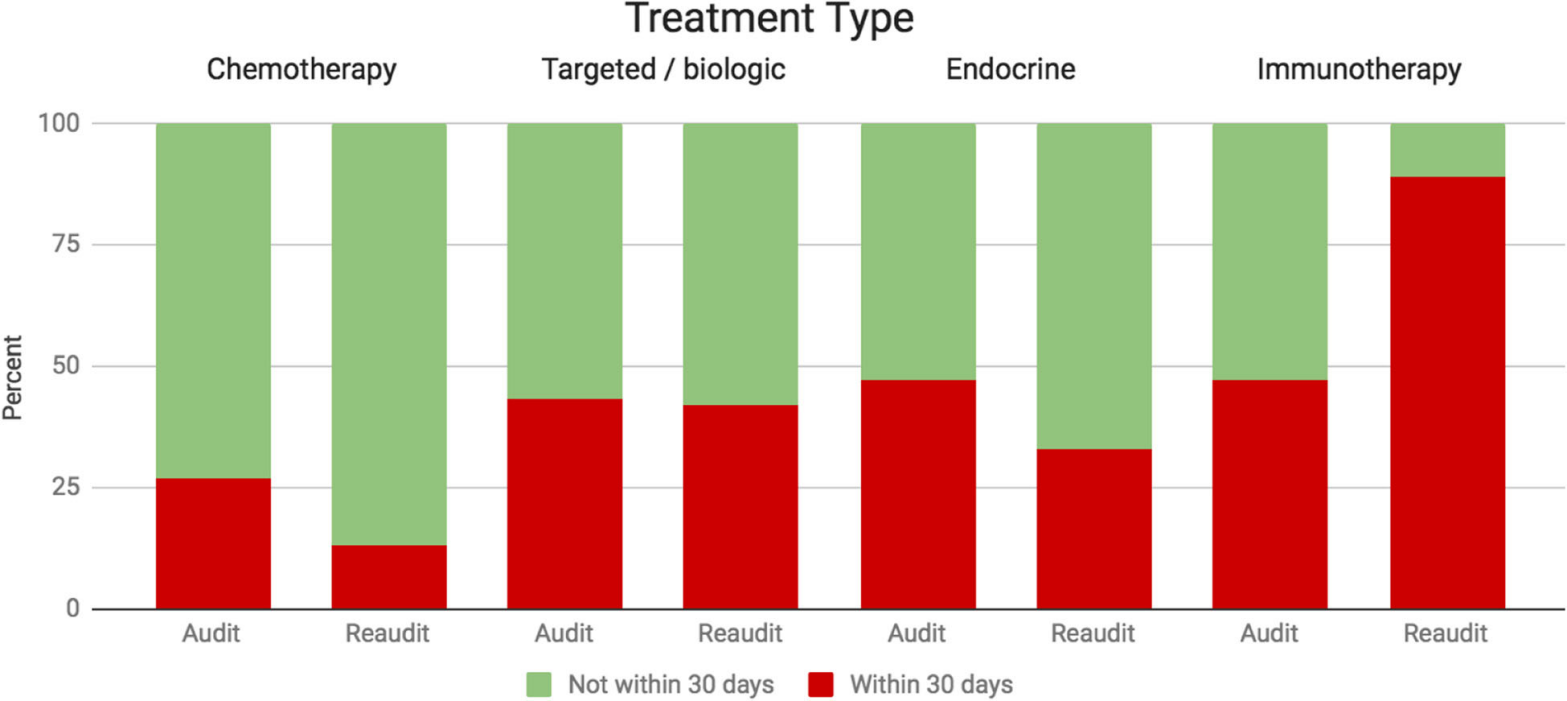
Comparison of rates of treatment between audit and re-audit period by treatment type

The treatments and aggressiveness of care in the re-audit period is summarised in Table 4 and Table 5. 8% of patients had not received any anticancer therapy whereas 33% had received one line of treatment, 33% had received two lines of treatment and 25% had received three or more lines of treatment. 19 patients of the total 75 deaths (25%) had received anticancer treatment within the last 30 days of life. 6 of 47 (13%) patients whose last anticancer treatment was chemotherapy received a dose within the last 30 days of life. This was substantially lower compared with the audit period. This indicator also reduced with respect to endocrine/hormonal treatments (33%) and remained stable with respect to targeted/biologics (42%). There was a substantial increase with regard to immune checkpoint inhibitors. 89% of patients, whose last treatment was an immune checkpoint inhibitor, received a dose within the last 30 days of life. This is demonstrated in Fig. 1. Of the total number of patients who received chemotherapy, treatment within the last 30 days of life represented 2% of patients.

**Table 5:** Treatment and aggressiveness of care in the re-audit period

	All Patients		Treatment within last 30 days		No treatment within last 30 days	
	Number	Percent	Number	Percent	Number	Percent
Last treatment type						
Chemotheratpy	47		6	13%	41	87%
Targeted/biologic	12		5	42%	7	58%
Endocrine/hormonal	6		2	33%	4	67%
Immunotherapy	9		8	89%	1	11%
Treatment intent						
Curative	6	8%	1	5%	5	9%
Palliative	69	92%	18	95%	51	91%
Parameters for aggressiveness of care						
Palliative care referral	55	80%	15	83%	40	78%
Palliative care referral beyond last 30 days	42	61%	12	67%	30	59%
More than one emergency presentations	6	9%	2	11%	4	8%
More than one hospitalisation	7	10%	3	17%	4	8%
Hospitalisation 14 or more days	15	22%	4	22%	11	22%
ICU admission	4	6%	1	6%	3	6%

#### Aggressiveness of care

92% of patients were treated with palliative intent. The rate of palliative care or hospice service referral was significantly improved to 80%. Late referrals were less frequent with 39% of referrals occurring in the last 30 days of life. Treatment within the last 30 days of life was again associated with more patients having more than one hospital admission (17% vs 8%). The rate of more than one ED presentation (11% vs 8%), ICU admission (6% vs 6%) and hospital admission greater than 14 days was similar (22% vs 22%).

## Discussion

Clinical audit is an essential part of clinical governance used to assess current performance and an important tool for practice improvement [29]. Audits have been found to have a small but potential important impact on professional practice [30]. This study reviewed 440 patient deaths and examined for patterns in SACT and other parameters of aggressiveness of care. A multifaceted quality improvement implementation plan was implemented and a re-audit of a further 75 patient deaths was conducted to assess the effect of this intervention. Components of the intervention have previously been proposed by other authors [10, 19]. The intervention was simple and can easily be replicated at other centres. The mechanism of the intervention is multifaceted. The initial education session raises awareness of this issue and reports on current performance. Regular reviews of all cases at morbidity and mortality meetings maintains awareness and allows repeated feedback to clinicians. Mandated repeat assessment of performance status and discussion of borderline cases at treatment commencement provide a decision point which can break up treatment inertia. Finally, simplifying referral processes to palliative care and hospice services supports patients, families and clinicians in symptom management, maximising the use of SACT and transitioning to the end of life period.

Overall, the patient population was as expected of a regional cancer centre with a predominance of solid tumour types representing the common cancer diagnoses and a broad spread of patient performance status. The rate of SACT within the last 30 days of life was 27% in the initial audit period and remained stable at 25% during the re-audit period. Review of the literature, as summarised in Table 1, did not find a published report to consider all treatment modalities, namely cytotoxic chemotherapy, endocrine and hormonal agents, targeted and biologic agents and immunotherapy. When comparing this figure to other published reports, it is important to note that this statistic is the number of deaths within the last 30 days of life as a proportion of all deaths of patients managed at the Goulburn Valley Health oncology department. A similar statistic in the Australian context was reported by Wein et al. at a rate of 26% but only included patients treated with palliative intent and did not include immunotherapy [21]. Other authors have reported treatment rates as a proportion of patient deaths between 4 and 38% [14, 16, 18, 21–23, 28].

The more commonly reported statistic is the number of deaths within 30 days of SACT as a proportion of all patients who received SACT. We calculated a comparable statistic where the number of deaths of patients who received chemotherapy within the last 30 days of life expressed as a percentage of all patients who received chemotherapy was initially 8% and fell substantially to 2% during the re-audit period. This compares well with reports from other centres and is in fact the lowest reported rate in Australasian region [11, 13, 19, 24–27].

This study is one of only a few to include immunotherapy in assessing mortality within 30 days of treatment. A recent study from New Zealand reported a rate of SACT within the last 30 days of life of 5.2% [11]. This included chemotherapy, targeted therapies and immunotherapy, but excluded endocrine / hormonal treatments. It should be noted that this was measured as a proportion of all patients who received SACT. Gilsch et al. conducted a retrospective review of 157 deceased patients treated with immune checkpoint inhibitors and reported that 27% received a dose within the last 30 days of life [4]. This is substantially lower than our rates of 47% in the audit period and 89% in the re-audit period.

SACT within the last 30 days of life with cytotoxic chemotherapy occurred in 27% and in higher proportions in non-chemotherapy treatments, specifically endocrine and hormonal agents 47%, targeted and biologic agents 43%, and immune checkpoint inhibitors 47%. Interestingly, this rate rose to 89% during the re-audit period with regard to immune checkpoint inhibitors whereas rates for endocrine / hormonal agents and targeted/biologic agents remained stable and the rate of chemotherapy use near the end of life fell to 13% (Fig. 1). This should be interpreted with caution due to the small patient numbers and short re-audit period. Possible explanations for these trends include the increasing availability and number of indications for immune checkpoint inhibitors over the recent period. Furthermore, non-chemotherapy treatments have a more tolerable side effect profile and may be more accepted by patients and clinicians when treatment decisions are being made. These factors may contribute to a shift in treatment modalities from chemotherapy towards immune checkpoint inhibitors. Also, the composition of tumour types between the audit and re-audit periods was different (Table 6) and may affect the types of treatments used.

**Table 6:** Top 10 most common tumour types in audit and re-audit periods

Audit			Re-audit		
Tumour type	Number	Percent	Tumour type	Number	Percent
Lung	87	20	Colorectal	14	19
Colorectal	62	14	Lung	10	13
Breast	42	10	Prostate	7	9
Prostate	40	9	Breast	6	8
Pancreas	34	8	Upper GI	5	7
Upper GI	29	7	Melanoma	5	7
Urothelial	18	4	Lymphoma	4	5
Gynaecological	15	3	Cholangiocarcinoma	4	5
Lymphoma	14	3	Head and neck	4	5
Melanoma	10	2	Gynaecological	3	4

**Fig. 2:**
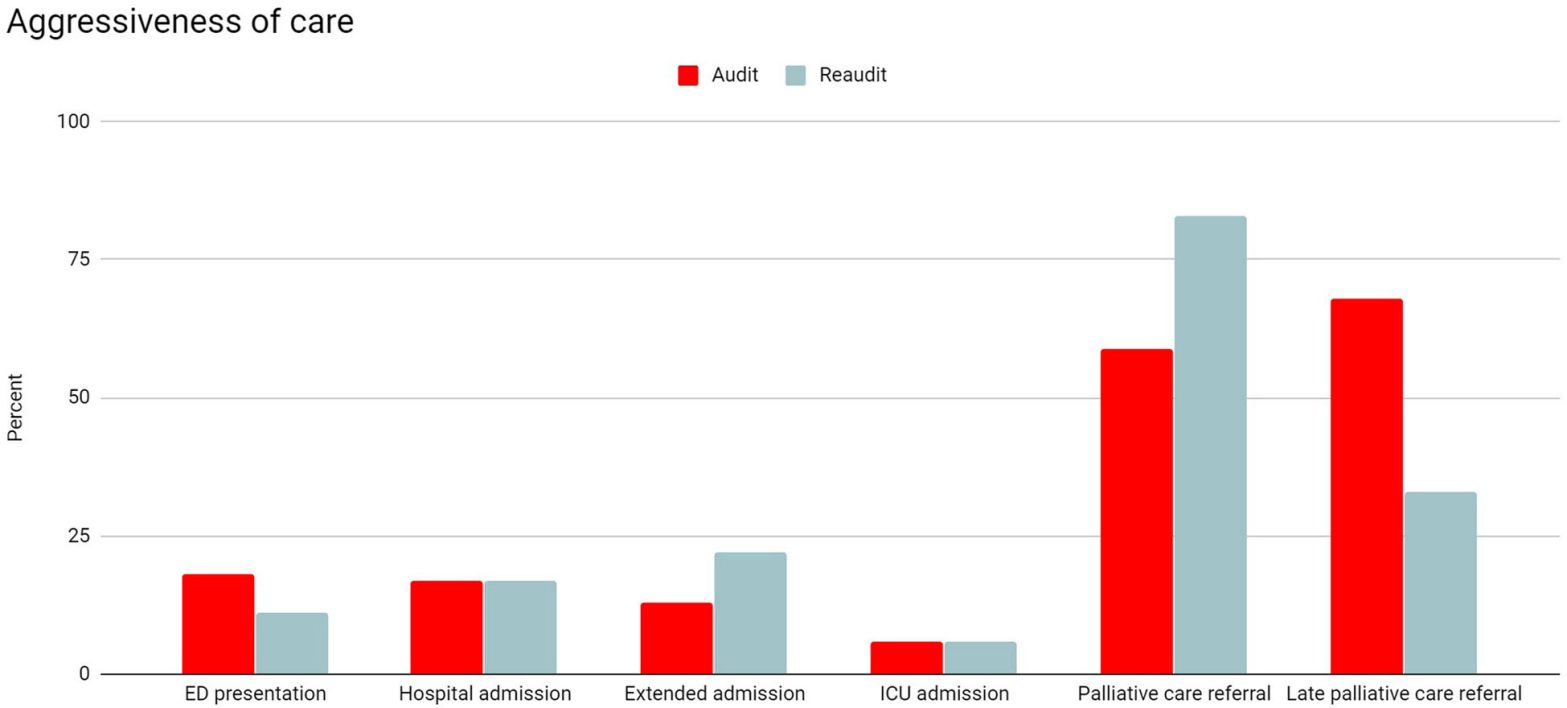
Comparison of indicators of aggressiveness of care between the audit and re-audit periods

When the other parameters of aggressiveness of care are examined, the re-audit period was notable for an increase in palliative care referrals (78% vs 65%) and decrease in late palliative care referrals (41% vs 58%). This did not seem to affect the other indicators of aggressiveness of care. Similar rates of more than one ED presentation, more than one hospitalisation and ICU admission were seen in the audit and re-audit period. The rate of hospital admission for more than 14 days rose from 14 to 22% after the intervention. This is demonstrated in Fig. 2. The association of treatment within the last 30 days of life and increased rates of more than one hospitalised was observed before and after the intervention but was not maintained with regard to more than one ED presentation or ICU admission.

Limitations to this project should be noted. Data has been collected in a retrospective manner. Data was collected from patient medical records which relies on complete and accurate documentation. Furthermore, patients in regional areas often have shared care between different centres and therefore a number of outcome events may not be captured in this data collection. The re-audit period was relatively shorter than the initial audit period and the observed trends in practice may attenuate over a longer period of time. The re-audit period was considered to have started immediately after the audit period which resulted in a short period of time before clinicians were fully exposed to the improvement implementation plan. The educational meeting and palliative care contacts were enacted within a week of the conclusion of the audit period. However, there may have been a learning curve period as clinicians gained repeated exposure and feedback from the regular case and mortality meetings. Nonetheless, these factors would likely have contributed to an underestimation of the effect of the improvement implementation plan.

## Conclusion

This study provides a contemporaneous benchmark for SACT and other parameters of aggressiveness of care within the last 30 days of life in an Australian regional setting. Importantly, the changing treatment paradigm with the increasing use of immune checkpoint inhibitors and other targeted agents is considered. It also establishes the components of a quality improvement implementation plan and demonstrates its impact on use of SACT and palliative care referral practices. Further research is required into the factors which affect the treatment decision making process in order to ensure quality of care.

## Data Availability

Data used from this study is available from the corresponding author on reasonable request.

## Abbreviations

ASCO: American Society of Clinical Oncology
ECOG: Eastern Cooperative Oncology Group
ED: emergency department
ESMO: European Society of Medical Oncology
ICU: Intensive care unit
NCEPOD: National Confidential Enquiry into Patient Outcome and Death
NSCLC: Non small cell lung cancer
QOL: Quality of life
SACT: Systemic anticancer therapy
